# Data on the weights, specific gravities and chemical compositions of potato (*Solanum tuberosum*) tubers for food processing from different areas of Hokkaido, Japan

**DOI:** 10.1016/j.dib.2017.03.009

**Published:** 2017-03-09

**Authors:** Hiroaki Sato, Ryosuke Koizumi, Yozo Nakazawa, Masao Yamazaki, Ryuichi Itoyama, Megumi Ichisawa, Junko Negichi, Rui Sakuma, Tadasu Furusho, Yoshimasa Sagane, Katsumi Takano

**Affiliations:** a Department of Food and Cosmetic Science, Faculty of Bioindustry, Tokyo University of Agriculture, 196 Yasaka, Abashiri, Hokkaido 099-2493, Japan; b Research Department, Research & Development Division, Calbee, Inc., 23-6 Kiyohara Kougyoudanchi, Utsunomiya, Tochigi 321-3231, Japan; c Department of Nutrition, Junior College of Tokyo University of Agriculture, 1-1-1 Sakuragaoka, Setagaya-ku, 156-8502 Tokyo, Japan; d Department of Applied Biology and Chemistry, Faculty of Applied Bioscience, Tokyo University of Agriculture, 1-1-1 Sakuragaoka, Setagaya-ku, Tokyo 156-8502, Japan

**Keywords:** Potato, Food processing, Chemical composition, Cultivar, Specific gravity

## Abstract

This data article provides the weights, specific gravities and chemical compositions (moisture, protein, fat, ash, and carbohydrate) of potato tubers, for food processing use, from the Tokachi, Kamikawa and Abashiri areas of Hokkaido, Japan. Potato tubers of four cultivars (‘Toyoshiro’, ‘Kitahime’, ‘Snowden’ and ‘Poroshiri’) were employed in the current study. The weights and specific gravities of potato tubers from each cultivar, harvested from three areas, were measured, and those of near average weight and specific gravity from each group were analyzed for their chemical composition. In this article, weight, specific gravity, and chemical composition data are provided in tables.

**Specifications Table**

**Table:** 

Subject area	Agricultural Science
More specific subject area	Food Chemistry
Type of data	Table
How data was acquired	A potato gauge, Model DPG400-C-B (Asahikawa-keiryoki, Asahikawa, Japan) was used to measure weights and specific gravities. Chemical compositions were measured using methods published by The Association of Official Analytical Chemists.
Data format	Raw, analyzed
Experimental factors	Potato tubers harvested in the Tokachi, Kamikawa and Abashiri areas of Hokkaido, Japan during autumn of 2014.
Experimental features	Weights, specific gravities and chemical compositions of potato tubers.
Data source location	Tokachi, Kamikawa and Abashiri areas, Hokkaido, Japan.
Data accessibility	Data are presented in this article.

**Value of the data**

• The data show the chemical compositions of potato tubers for food processing use from different areas of Hokkaido, Japan.
• The data presented form a reference for estimating the nutritional value of potato tubers.
• The data presented are available for use when comparing the chemical compositions of potato tubers harvested from other area.

## 1 Data

This article presents data on weight distribution (Table 1), specific gravity distribution (Table 2), and chemical composition (Table 3) of potato tubers of four cultivars, namely ‘Toyoshiro’, ‘Kitahime’, ‘Snowden’ and ‘Poroshiri’. Potato tubers from each cultivar were harvested at farms in the Tokachi, Kamikawa and Abashiri areas of Hokkaido, Japan.

## 2 Experimental design, materials and methods

We selected the four cultivars that are most widely farmed for food processing purposes. ‘Toyoshiro’ is the most popular cultivar and provides the highest yield for processing purposes in Japan [1]. ‘Kitahime’, ‘Snowden’ and ‘Poroshiri’ are predominantly used to produce potato chips. These four cultivars were sourced from Tokachi, Kamikawa, and Abashiri. The weights and specific gravities of the potato tubers were measured. Tubers of around average weight and specific gravity from each cultivar were then selected for chemical composition analysis.

### 2.1 Materials

Four potato cultivars (‘Toyoshiro’, ‘Kitahime’, ‘Snowden’ and ‘Poroshiri’) were used in this study. These tubers were harvested from farms in the Tokachi, Kamikawa and Abashiri areas of Hokkaido, Japan, during the autumn of 2014.

### 2.2 Measurements of weight and specific gravity

Each individual tuber, from 20-kg sets of potato tubers of each cultivar, were weighed using a potato gauge (GPG400-C-B, Asahikawa-Keiryoki, Asahikawa, Japan), both in air and water, according to the method described by Murphy and Goven [2], in order to calculate the specific gravity of each tuber. Twenty potato tubers, having weights and specific gravities around the average value for each group, were selected and assayed for their chemical compositions.

### 2.3 Determination of chemical composition

Before preparing freeze-dried samples, the moisture content of each fresh potato tuber (within a week of harvesting) was measured. The tubers were peeled, vacuum packaged, and frozen by immersing in acetone at −40°C. The samples were subsequently freeze-dried using a freeze dryer. Freeze-dried samples were analyzed for their moisture, protein, fat and ash content, according to methods published by the Association of Official Analytical Chemists (AOAC) (1990) [3], [4]. Carbohydrate content was determined by difference calculations [100−(moisture + protein + fat + ash)%].

## Figures and Tables

**Table 1:** Weight distribution of potato tubers as percentage of total yield.

Tuber weight (g)	‘Toyoshiro’			‘Kitahime’		
	Tokachi	Kamikawa	Abashiri	Tokachi	Kamikawa	Abashiri
25–50	0.7	0.0	5.3	0.0	0.0	2.5
50–75	9.9	20.6	16.4	9.3	18.9	6.6
75–100	13.8	27.4	27.5	15.0	28.4	6.6
100–125	21.7	21.7	24.6	25.0	20.3	15.7
125–150	17.8	14.3	12.9	20.0	20.3	14.0
150–175	13.2	8.6	5.3	12.9	8.1	15.7
175–200	7.9	5.7	5.8	7.9	3.4	9.9
200–225	7.9	0.6	1.2	5.7	0.0	14.9
225–250	4.6	0.0	1.2	0.7	0.7	6.6
250–275	2.0	0.6	0.0	2.1	0.0	4.1
275–300	0.7	0.6	0.0	0.7	0.0	3.3
300-	0.0	0.0	0.0	0.7	0.0	0.0
Tuber weight (g)	‘Snowden’			‘Poroshiri’		
	Tokachi	Kamikawa	Abashiri	Tokachi	Kamikawa	Abashiri

25–50	3.0	21.5	0.0	0.7	n.d.	0.0
50–75	18.8	30.7	4.3	12.0	n.d.	3.2
75–100	30.9	27.0	12.0	13.4	n.d.	15.3
100–125	24.2	12.9	13.7	26.8	n.d.	16.9
125–150	10.3	4.9	15.4	16.9	n.d.	17.7
150–175	6.7	1.2	13.7	16.2	n.d.	16.9
175–200	1.8	0.0	14.5	9.9	n.d.	12.9
200–225	1.8	1.8	12.0	1.4	n.d.	8.9
225–250	1.8	0.0	6.8	2.1	n.d.	5.6
250–275	0.6	0.0	4.3	0.7	n.d.	1.6
275–300	0.0	0.0	1.7	0.0	n.d.	0.8
300-	0.0	0.0	1.7	0.0	n.d.	0.0

n.d.: No data

The value in the first column is greater than the number given, but less than the number in the following line.

**Table 2:** Specific gravity distribution of potato tubers as percentage of total yield.

Specific gravity (g/cm^3^)	‘Toyoshiro’			‘Kitahime’		
	Tokachi	Kamikawa	Abashiri	Tokachi	Kamikawa	Abashiri
1.055–1.060	0.0	0.0	0.0	0.0	0.0	0.8
1.060–1.065	0.0	0.0	0.0	1.4	0.0	0.8
1.065–1.070	2.0	0.6	0.0	7.1	2.0	0.8
1.070–1.075	5.9	8.6	0.0	15.0	4.7	8.3
1.075–1.080	14.5	18.3	1.2	20.7	8.1	9.1
1.080–1.085	15.1	40.6	6.4	27.9	16.9	20.7
1.085–1.090	21.7	20.6	18.7	17.9	25.0	27.3
1.090–1.095	22.4	9.7	26.9	6.4	25.0	20.7
1.095–1.100	11.8	1.7	29.2	3.6	14.2	9.9
1.100–1.105	3.9	0.0	12.3	0.0	4.1	1.7
1.105–1.110	2.6	0.0	4.7	0.0	0.0	0.0
1.110–1.115	0.0	0.0	0.6	0.0	0.0	0.0
1.115–1.120	0.0	0.0	0.0	0.0	0.0	0.0
1.120-	0.0	0.0	0.0	0.0	0.0	0.0
Specific gravity (g/cm^3^)	‘Snowden’			‘Poroshiri’		
	Tokachi	Kamikawa	Abashiri	Tokachi	Kamikawa	Abashiri

1.055–1.060	0.0	0.0	0.9	0.0	n.d.	0.0
1.060–1.065	0.0	0.0	0.9	0.7	n.d.	0.0
1.065–1.070	0.0	0.0	4.3	2.1	n.d.	0.8
1.070–1.075	0.0	1.2	17.1	7.7	n.d.	1.6
1.075–1.080	3.0	4.3	22.2	12.7	n.d.	10.5
1.080–1.085	10.3	12.3	20.5	26.1	n.d.	18.5
1.085–1.090	27.3	25.2	20.5	31.7	n.d.	29.0
1.090–1.095	32.7	29.4	7.7	12.0	n.d.	25.8
1.095–1.100	19.4	21.5	4.3	5.6	n.d.	7.3
1.100–1.105	5.5	6.1	1.7	1.4	n.d.	4.0
1.105–1.110	1.8	0.0	0.0	0.0	n.d.	0.8
1.110–1.115	0.0	0.0	0.0	0.0	n.d.	0.0
1.115–1.120	0.0	0.0	0.0	0.0	n.d.	0.0
1.120-	0.0	0.0	0.0	0.0	n.d.	1.6

n.d.: No data

The value in the first column is greater than the number given, but less than the number in the following line.

**Table 3:** Chemical composition of potato tubers of average size.

	‘Toyoshiro’			‘Kitahime’		
	Tokachi	Kamikawa	Abashiri	Tokachi	Kamikawa	Abashiri
Moisture (%)	79.34	80.71	78.09	81.07	79.09	79.75
Protein (%)	1.69	1.92	1.67	1.88	1.37	1.70
Fat (%)	0.04	0.04	0.06	0.04	0.05	0.04
Ash (%)	0.88	0.93	1.02	0.99	1.03	1.03
Carbohydrate (%)	18.05	16.39	19.16	16.02	18.46	17.47
	‘Snowden’			‘Poroshiri’		
	Tokachi	Kamikawa	Abashiri	Tokachi	Kamikawa	Abashiri

Moisture (%)	78.55	79.02	82.98	80.47	n.d.	79.67
Protein (%)	1.67	1.59	1.84	1.59	n.d.	1.78
Fat (%)	0.04	0.03	0.04	0.05	n.d.	0.05
Ash (%)	0.98	0.97	0.90	0.91	n.d.	0.98
Carbohydrate (%)	18.77	18.38	14.25	16.98	n.d.	17.52

n.d.: No data
